# Application of machine learning in predicting perioperative neurocognitive disorders in elderly patients: the impact of sarcopenia-related features

**DOI:** 10.3389/fmed.2025.1604333

**Published:** 2025-08-18

**Authors:** Zhengyu Qian, Xiaochu Wu, Kunyang He, Kaijie Lin, Xiaobei Luo, Tianyao Zhang

**Affiliations:** ^1^School of Clinical Medicine, Chengdu Medical College, Chengdu, China; ^2^The First Affiliated Hospital of Chengdu Medical College, Chengdu, China; ^3^National Clinical Research Center for Geriatrics, West China Hospital, Sichuan University, Chengdu, Sichuan, China; ^4^Suzhou Medical College, Soochow University, Suzhou, Jiangsu, China

**Keywords:** machine learning, sarcopenia, postoperative cognitive dysfunction, SHapley Additive exPlanations, perioperative neurocognitive disorders

## Abstract

**Background:**

Older surgical patients present with diverse clinical profiles, yet research indicates a significant correlation between sarcopenia-related features and the incidence of perioperative neurocognitive disorder (PND). The integration of machine learning techniques offers a promising avenue for identifying older surgical patients at elevated risk of PND, particularly those exhibiting sarcopenia-associated characteristics. This approach enhances preoperative risk stratification and patient selection, thereby improving the precision of clinical management and treatment decisions.

**Methods:**

Data were collected from patients undergoing non-cardiac surgery at the First Affiliated Hospital of Chengdu Medical College to develop and validate a predictive model. Five machine learning models—Support Vector Machine (SVM), Extreme Gradient Boosting (XGBoost), Gradient Boosting Machine (GBM), Adaptive Boosting (AdaBoost), and Random Forest—were constructed to evaluate the risk of PND in older surgical patients. Sarcopenia-related features were incorporated as key variables in these models. The SHapley Additive exPlanations (SHAP) method was subsequently utilized to interpret the most effective model.

**Results:**

A total of 443 patients were included in the study. Among the five models, AdaBoost performed best, achieving an AUC of 0.95. The six most important features identified by SHAP were 6-meter walking speed, preoperative MMSE score, maximum grip strength, appendicular skeletal muscle mass, and sarcopenia assessment age. These results demonstrate AdaBoost's excellent predictive performance, with high interpretability and reliability.

**Conclusion:**

Machine learning models, particularly AdaBoost integrated with SHAP, show significant potential in predicting PND in older surgical patients. The model's ability to clarify the impact of sarcopenia-related features enhances its clinical utility in preoperative risk assessment.

## 1 Introduction

Perioperative Neurocognitive Disorders (PND) encompass postoperative cognitive impairments such as memory, attention, and language deficits, predominantly observed in older surgical patients (1, 2). These disorders often manifest initially as postoperative delirium (POD) or emergence agitation, evolving into more chronic forms such as postoperative cognitive dysfunction (POCD) (3). PND is associated with prolonged hospitalization, increased postoperative complications, and significantly diminishes the quality of life, imposing substantial burdens on families and societal resources (4).

A global increase in the olderly population (5) corresponds with heightened surgical demand (6), notably among those over 60, with dementia symptoms and PND rates reaching up to 65% (7). Research indicates that 41.4% of patients older than 60 suffer from PND post-surgery, with a notable percentage not fully recovering within 3 months (8), underscoring the necessity for preemptive risk management in anesthesia.

Sarcopenia, defined by progressive loss of skeletal muscle mass and strength, occurs in up to 33% of individuals over 65 and significantly correlates with PND (9). This condition contributes to cognitive decline through mechanisms such as reduced neurogenesis and lower levels of critical neurotrophic factors, including brain-derived neurotrophic factor (BDNF) (10). Furthermore, sarcopenia is associated with prolonged postoperative recovery, heightened susceptibility to complications, including increased infection risk and extended hospitalization periods. Notably, one study documented a 15% increase in PND incidence among patients exhibiting sarcopenia prior to surgery (11). An animal study also underscored reduced skeletal muscle content as a significant preoperative risk factor for PND (10). Therefore, factors affecting muscle mass, such as inflammation and insulin resistance, are closely associated with PND, suggesting that sarcopenia-related features could be critical risk factors for PND in the older adult (12, 13).

Machine Learning (ML) advancements have significantly transformed medical diagnostics and treatment planning. ML algorithms surpass traditional logistic regression in detecting intricate non-linear relationships and managing complex datasets, thereby enhancing prediction accuracy for clinical outcomes. Current research predominantly targets older surgical patients undergoing cardiac and non-cardiac surgeries, with ML models evaluating factors such as type of surgery, preoperative cognitive status, and anesthesia methods to provide a reliable level of predictive accuracy (14, 15). Despite progress, research tailored to high-risk elderly populations with specific risk profiles, such as those with sarcopenia, remains limited.

This study develops an ML-based predictive model targeting sarcopenia-related risk factors, aiming to optimize perioperative management and enhance postoperative outcomes for older surgical patients. By conducting detailed preoperative assessments, the proposed model intends to predict PND risk accurately, thus refining management strategies and enhancing the postoperative quality of life for these high-risk groups.

## 2 Materials and methods

### 2.1 Ethical approval

This study adhered to the principles of the Declaration of Helsinki and was approved by the Medical Ethics Committee of the First Affiliated Hospital of Chengdu Medical College (Approval No: 2021CYFYIRB-BA-67-01) on November 28, 2021. All participants provided written informed consent prior to their inclusion in the study.

### 2.2 Study design and population

In this prospective cohort study, clinical electronic medical records and relevant data were systematically collected from patients undergoing surgical procedures at the First Affiliated Hospital of Chengdu Medical College via the iMedical system between March 1, 2022, and February 28, 2023. A total of 480 patients were initially enrolled, of whom 443 satisfied the inclusion criteria for model development and internal validation. Preoperative demographic and clinical characteristics were documented at the time of patient admission, while perioperative treatment data and postoperative clinical outcomes were meticulously recorded thereafter. All predictive variables were gathered prior to the onset of perioperative neurocognitive disorder (PND) to facilitate the adjustment of perioperative management strategies by anesthesiologists, based on real-time risk evaluations.

Inclusion criteria included patients aged ≥60 years undergoing elective non-cardiac surgery, with specific sarcopenia-related indicators (e.g., appendicular skeletal muscle mass) collected. Exclusion criteria comprised inability to complete gait speed and grip strength assessments; presence of severe systemic diseases affecting major organs (heart, lungs, liver, kidneys); visual, hearing, or communication impairments hindering questionnaire completion or examination cooperation; history of significant preoperative mental illness or comprehensive neurological disorders, such as cognitive impairment; and cases with severe data loss in the study.

### 2.3 Diagnostic criteria for PND and sarcopenia

Given the absence of a universal diagnostic standard for PND, this study defined PND as a decrease of ≥2 points in the Mini-Mental State Examination (MMSE) score 3 days postoperatively compared to the preoperative score (16). MMSE scores were measured 1 day before surgery and on the third postoperative day for all cases included in this study. The diagnostic criteria for sarcopenia were based on the 2019 standards established by the Asian Working Group for Sarcopenia (AWGS) (17): 1. Muscle strength: grip strength (men < 28 kg, women < 18 kg); 2. Physical function: 6-meter walking speed < 1.0 m/s; 3. Appendicular skeletal muscle mass: BIA (men < 7.0 kg/m^2^, women < 5.7 kg/m^2^).

### 2.4 Feature selection and model development

In the dataset used in this study, only the variable “age” had missing values, affecting two patients (0.45% of the total sample); all other variables were complete. Outliers in continuous variables were identified using the interquartile range (IQR) method and removed to reduce skewness. Continuous variables were normalized using min-max scaling for comparability across models. Missing values were imputed using multivariate imputation by chained equations (MICE, 4 iterations) (38).

Initially, we performed univariate regression analysis on each variable. For categorical variables, differences between the two groups were compared using Fisher's exact test or chi-square test, depending on sample size and expected frequency. For continuous variables following a normal distribution, an independent samples *t*-test was used for inter-group comparisons; for continuous variables not following a normal distribution, the Mann-Whitney *U*-test (rank-sum test) was employed. The Shapiro-Wilk test was used to assess the normality of the continuous variables to determine the appropriate statistical methods. A *P*-value < 0.05 was considered statistically significant.

We conducted logistic regression analysis on important variables from the univariate analysis to identify significant features for subsequent evaluation and construction of ML predictive models. The original dataset was randomly divided into a test set (30%) and a training set (70%). The training set was used to train the ML models, while the test set was used to adjust model parameters and evaluate model performance. All significant features identified by logistic regression were incorporated into five ML models to predict PND. These models included Support Vector Machine (SVM), Extreme Gradient Boosting (XGBoost), Gradient Boosting Machine (GBM), Adaptive Boosting (AdaBoost), and Random Forest. To avoid overfitting, we used a grid search algorithm with 10-fold cross-validation to optimize the training set and find the best hyperparameters for training our ML models, ensuring good generalization ability. Stratified sampling was used during cross-validation to maintain the class distribution between PND and non-PND cases, thereby mitigating imbalance without the need for synthetic oversampling.

For the AdaBoost model, we performed hyperparameter tuning using grid search with 10-fold cross-validation. The tuning grid included 27 combinations of the number of iterations (50, 100, 150), maximum tree depth (1, 2, 3), and learning rate (ν = 0.1, 0.5, 1).

While AUC was the primary metric for model evaluation, F1 score was used as the guiding metric during hyperparameter tuning, as it provides a more balanced assessment under class imbalance. The best-tuned model achieved an AUC of 0.952, substantially outperforming the default AdaBoost configuration (AUC = 0.910) under identical validation conditions.

### 2.5 Model evaluation and interpretation

To evaluate the robustness of the model with respect to missing data imputation, we conducted a sensitivity analysis comparing the full dataset (*n* = 443) and the complete-case dataset (*n* = 441). Excluding the two patients with missing age values led to a decrease in AUC from 0.952 to 0.892. Both excluded cases were PND-positive and exhibited atypical profiles, including high MMSE scores and good physical performance, suggesting that their inclusion enhanced model discrimination. These findings support the stability of the AdaBoost model and justify the use of the imputed dataset for subsequent model training and interpretation. Details are provided in Supplementary Table S1 and Supplementary Figure S1.

Our evaluation of model performance focused on discrimination and calibration. We used the area under the receiver operating characteristic curve (AUROC) to reflect discriminative ability and described the statistical differences in AUROC using the Delong test (18). Additionally, when evaluating the performance of machine learning models, we calculated accuracy, precision, recall, and F1 score, and referenced the Youden index to optimize and select the best cutoff value to balance sensitivity and specificity. These metrics comprehensively measure model performance from different perspectives, ensuring that we can identify the strengths and weaknesses of the models in various application scenarios and make specific improvements to optimize overall performance. The interpretability of machine learning models has always been challenging. To further explain the impact and contribution of each feature variable to the final model, we used the SHAP method to interpret the best-performing black-box model (19). This ranking process is based on the mean absolute SHAP values of all individuals. These interpretability techniques were implemented in R using the “iml” package version 0.11.3.

### 2.6 Language editing assistance

During the preparation of this manuscript, we used ChatGPT to assist with language editing and to improve the readability of the text. All scientific content and data analysis were conducted independently by the authors.

## 3 Results

### 3.1 Patient characteristics

A total of 443 older surgical patients were included in this study, of whom 121 developed PND, resulting in an incidence rate of 27.3%. The Activities of Daily Living (ADL) scale is commonly used to assess an individual's ability to perform basic and instrumental activities, with a score of 100 indicating good self-care ability. The Instrumental Activities of Daily Living (IADL) scale assesses the ability to perform more complex daily activities. The total IADL score ranges from 0 to 8, with a score of 8 indicating that the individual can independently complete all activities, demonstrating a high level of self-care ability. A frailty score >0 indicates some degree of frailty or a pre-frailty state. Detailed clinical characteristics are presented in Table 1.

**Table 1:** *P*-values were obtained by comparing the PND and non-PND groups using the Mann-Whitney *U*-test a; Chi-square test or Fisher's exact test b; independent samples *t*-test c; Variables marked with ^*^ were included in the logistic regression model (*P* < 0.05).

**Variables**	**PND (*N* = 121, 27.3%)**	**Non-PND (*N* = 322, 72.6%)**	***P*-values**
**Demographics**			
Age, year, median (IQR)	73 (69–73)	71 (68–75)	0.020^a*^
**Gender**, ***n*** **(%)**			0.383^b*^
Male	67 (52.9)	187 (41.9)	
Female	54 (47.1)	135 (58.1)	
**Activities of daily living**, ***n*** **(%)**			
ADL score (< 100)	14 (11.6)	30 (9.3)	< 0.001^b*^
IADL score (< 8)	52 (43.0)	142 (44.1)	< 0.001^b*^
Frailty level (>0)	60 (49.6)	126 (39.1)	0.139^b^
Frailty score (>0)	72 (59.5)	165 (51.2)	0.002^a*^
**Preoperative cognitive function variables, median (IQR)**			
Preoperative orientation	9 (7.0–10)	8 (7.0–10.0)	0.032^b*^
Preoperative immediate memory	3 (3.0–3.0)	3 (3.0–3.0)	< 0.001^b*^
Preoperative attention and calculation	5 (3.0–5.0)	5 (4.0–5.0)	< 0.001^b*^
Preoperative delayed memory	3 (3.0–3.0)	3 (3.0–3.0)	0.482^b*^
Preoperative language ability	7 (5.0–8.0)	7 (5.0–8.0)	0.605^b*^
Preoperative visuospatial ability	1 (0.0–1.0)	1 (0.0–1.0)	< 0.001^b*^
Preoperative cognitive impairment present	0 (0.0–0.0)	0 (0.0–0.0)	1^b*^
Preoperative MMSE score	26 (24.0–28.0)	26 (24.0–27.0)	0.15 ^b*^
**Physical function variables**			
Mini Nutritional Assessment, median (IQR)	13 (11.0–14.0)	13 (11.0–14.0)	0.436^a^
6-meter walking speed, median (IQR)	0.81 (0.62–0.94)	0.95 (0.79–1.10)	< 0.001^c*^
Maximum grip strength, median (IQR)	23.5 (17.2–26.9)	24.9 (20.33–31.48)	< 0.001^c*^
Appendicular skeletal muscle mass, median (IQR)	6.5 (5.80–7.31)	7.0 (6.23–7.77)	< 0.001^c*^
Sarcopenia assessment, *n* (%)	58 (47.9)	65 (20.2)	< 0.001^b*^
**Anesthesia and surgery-related variables**			
ASA classification, *n* (%)			0.367^b^
I	69 (57.0)	183 (56.8)	
II	51 (42.1)	139 (43.2)	
III	1 (0.09)	0 (0.0)	
Use of dexmedetomidine, *n* (%)	37 (30.6)	103 (32.0)	0.865^b*^
Combined nerve block, *n* (%)	52 (43.0)	144 (44.7)	0.824^b^
Type of anesthesia, *n* (%)			< 0.001^b*^
Local anesthesia	3 (2.5)	1 (0.04)	
Subarachnoid anesthesia	32 (26.5)	71 (22.0)	
General anesthesia	86 (71.0)	250 (77.6)	
Use of analgesic pump, *n* (%)	63 (52.1)	182 (56.5)	< 0.001^b*^
Duration of surgery, min, median (IQR)	106 (64–185)	110 (63–210)	0.564^a*^
Duration of anesthesia, min, median (IQR)	150 (101–237)	150 (100–264)	0.455^a*^
Blood loss, ml, median (IQR)	20 (10–50)	20 (10–50)	0.999
Fluid infusion, ml, median (IQR)	1100 (1000–2050)	1200 (1000–2100)	0.447
**Medical history**, ***n*** **(%)**			
Diabetes	37 (30.5)	85 (26.4)	0.448^b*^
Hypertension	58 (47.9)	127 (39.4)	< 0.001^b*^

### 3.2 Model evaluation

**Figure 1:**
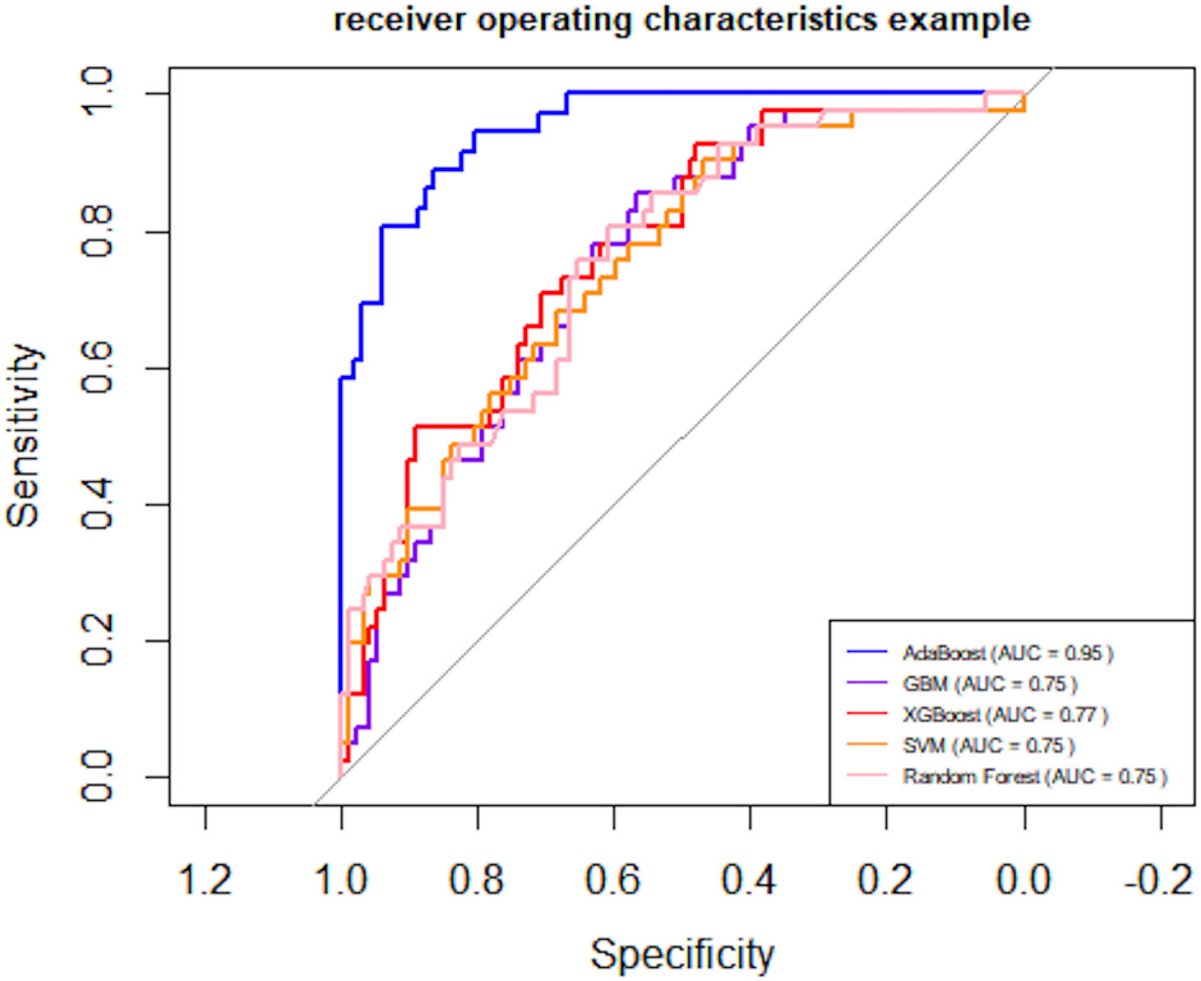
Receiver Operating Characteristic (ROC) curves. AUC (Area Under the Curve) for SVM (Support Vector Machine), XGBoost (Extreme Gradient Boosting), GBM (Gradient Boosting Machine), AdaBoost (Adaptive Boosting), and Random Forest.

Following logistic regression analysis, we incorporated 22 variables into the model, including: age, ADL scale, IADL scale, frailty score, preoperative orientation, preoperative immediate memory, preoperative attention and calculation, preoperative delayed memory, preoperative language ability, preoperative visuospatial ability, preoperative presence of cognitive impairment, preoperative MMSE score, 6-meter walking speed, maximum grip strength, appendicular skeletal muscle mass, sarcopenia assessment, hypertension, anesthesia type, dexmedetomidine usage, analgesia pump usage, surgery duration, and anesthesia duration. Five algorithms were employed: SVM, XGBoost, GBM, AdaBoost, and Random Forest. The results indicated that the AdaBoost model achieved the highest area under the receiver operating characteristic curve (AUC) on the test set (Figure 1). Moreover, the AdaBoost model outperformed the other models in all other performance metrics. The details of the specific model parameters developed using different algorithms are shown in Table 2.

**Table 2:** Performance Metrics of Different Machine Learning Models for PND.

**Model**	**Accuracy (95% CI)**	**Precision (95% CI)**	**Recall (95% CI)**	**F1 Score (95% CI)**
AdaBoost	0.88 (0.82-0.93)	0.89 (0.83-0.95)	0.95 (0.91-0.99)	0.92 (0.87-0.97)
XGBoost	0.77 (0.70-0.84)	0.79 (0.72-0.86)	0.90 (0.85-0.95)	0.84 (0.78-0.90)
GBM	0.69 (0.61-0.77)	0.69 (0.62-0.77)	0.99 (0.97-1.01)	0.82 (0.75-0.88)
SVM	0.70 (0.62-0.78)	0.70 (0.62-0.78)	0.99 (0.97-1.01)	0.82 (0.75-0.88)
RF	0.74 (0.66-0.81)	0.75 (0.67-0.82)	0.93 (0.89-0.98)	0.83 (0.77-0.89)

To provide a conventional benchmark, we additionally evaluated a logistic regression model using the same dataset and 10-fold cross-validation strategy. The model yielded an AUC of 0.708 (95% CI: 0.653–0.763), which was substantially lower than the performance of the AdaBoost model (AUC = 0.952, 95% CI: 0.918–0.985). This result highlights the added predictive value of the machine learning approach over traditional linear modeling techniques.

### 3.3 Model interpretation

**Figure 2:**
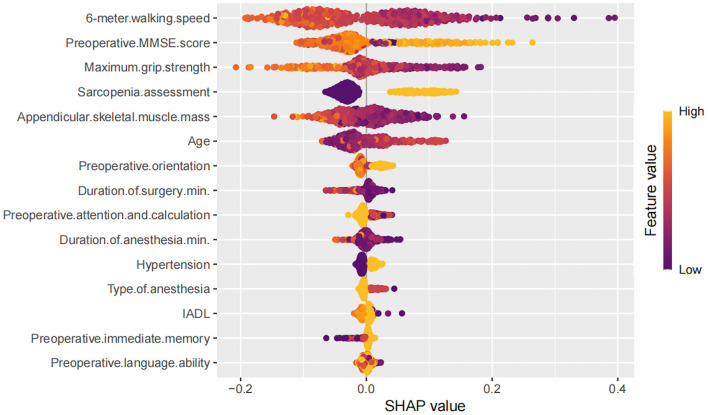
Decision path diagram showing the contributions of the top 15 features affecting Perioperative Neurocognitive Disorders.

To better understand the relationship between the model and the data, we used the SHAP method to visually interpret the best performing AdaBoost model, illustrating how each variable affects the occurrence of PND. SHAP values were used to explain the 15 assessed risk factors. As shown in Figure 2, the SHAP values on the *X*-axis represent a unified metric that shows how each feature influences the model's outcome. For each feature, the impact on the outcome is represented by colored dots, with high feature values indicated by yellow and low feature values by purple. The *Y*-axis lists the 15 factors that are statistically significant for the PND outcome.

Higher values of features (points closer to yellow) such as maximum grip strength, appendicular skeletal muscle mass, preoperative MMSE score, and 6-meter walking speed were generally associated with negative SHAP values, indicating a protective effect against PND. In contrast, features like older age, longer surgery and anesthesia duration, hypertension, general anesthesia, and sarcopenia assessment showed positive SHAP contributions, indicating increased risk. Other variables (e.g., IADL, preoperative language ability, immediate memory) exhibited minimal or inconsistent SHAP effects.

To further assess non-linear and interaction effects revealed by the SHAP analysis, we examined the dependence plot for age. The SHAP value for age showed a non-monotonic trend—rising up to ~80 years, then plateauing or slightly declining—possibly reflecting survivor bias or physiological adaptation in the oldest patients.

**Figure 3:**
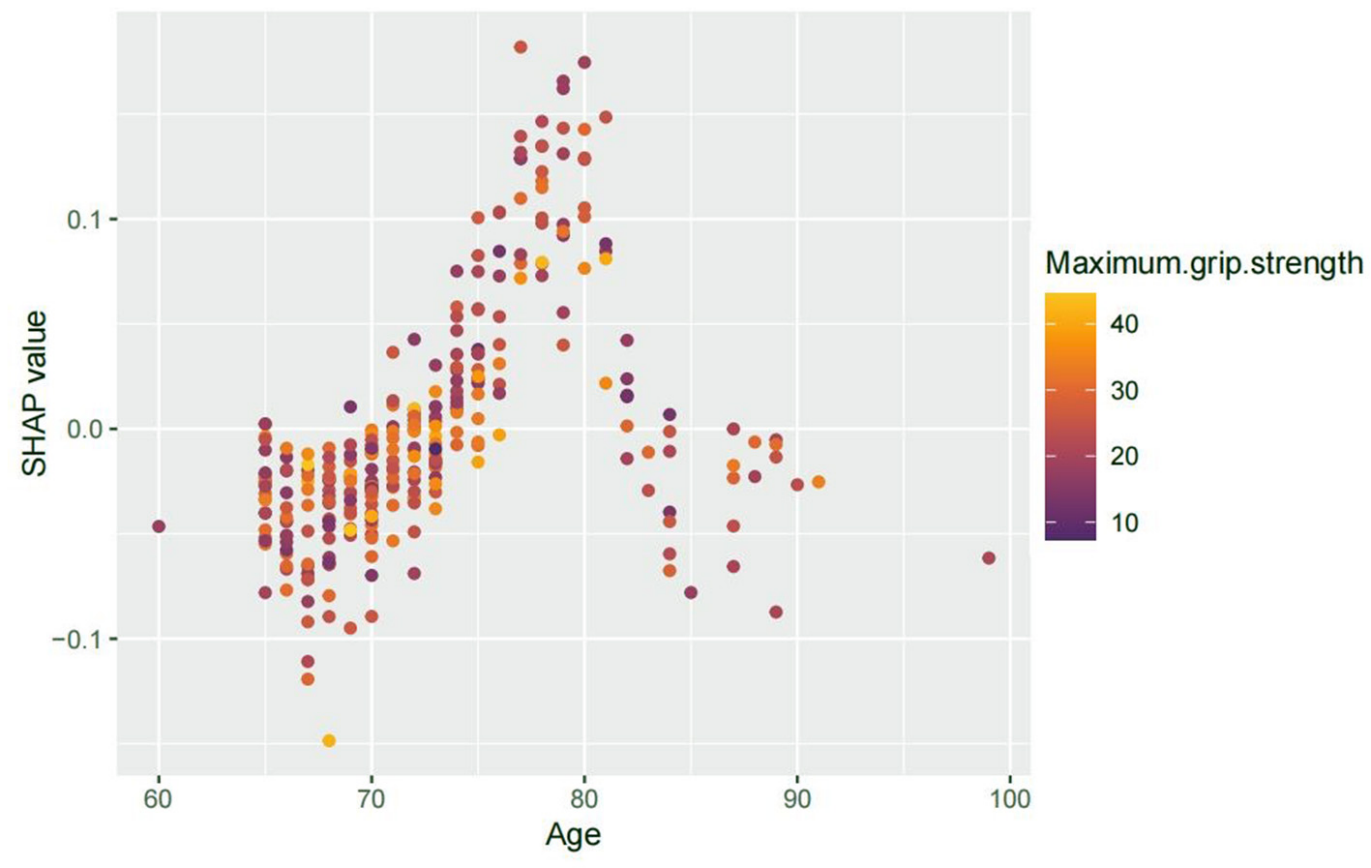
SHAP interaction plot showing age vs. PND risk, colored by grip strength. Advancing age was associated with increased SHAP values for PND risk, peaking around 80 years. While not uniformly distributed, patients with lower grip strength appeared to show relatively elevated SHAP values for age, suggesting a potential interaction in which reduced muscle strength may enhance age-related vulnerability to PND.

We also explored the interaction between age and grip strength (Figure 3). Patients with lower grip strength tended to show relatively higher SHAP values for age, suggesting a possible interaction pattern. While this trend was not consistent across all data points, the visual distribution implies that reduced muscle strength may be associated with increased age-related vulnerability to PND. This observation highlights the potential role of sarcopenia not only as an independent risk factor but also as a contributor to age-related susceptibility.

### 3.4 Comparative AUC performance and DeLong tests

To further quantify performance differences, we compared the AUC values of all models using the DeLong test. As shown in Table 3, AdaBoost significantly outperformed all other models (*P* < 0.001). The logistic regression model yielded an AUC of 0.708 (95% CI: 0.653–0.763), whereas the AUCs for XGBoost, GBM, SVM, and Random Forest ranged from 0.746 to 0.775.

**Table 3:** AUC values represent model discrimination performance in the test set.

**Model**	**AUC**	***P*-value vs. AdaBoost**
AdaBoost	0.95	-
XGBoost	0.77	0.0002
GBM	0.75	2.7e-05
SVM	0.75	4.6e-05
RF	0.75	1.6e-05

*P*-values were obtained by comparing each model with the AdaBoost model using the DeLong test.

## 4 Discussion

In this retrospective cohort study, we developed a model to predict the risk of PND among older surgical patients, specifically incorporating sarcopenia-related indicators. We used 23 important features to train and validate the model using SVM, XGBoost, GBM, AdaBoost, and Random Forest. Among the different models compared, the AdaBoost model showed the best predictive power, with an AUC of 0.95, precision of 0.89, sensitivity of 0.95.

Data imbalance can significantly affect the accuracy of predictive models. In our study cohort of elderly individuals, the relatively low prevalence of PND (incidence rate of 27.3%) created a notable disparity between normal and affected samples. This class imbalance means that a model could theoretically achieve an accuracy of 88% by predicting all outcomes as normal. However, such predictions are misleading, as they fail to adequately identify PND cases, leading to a bias toward predicting normal outcomes and reducing precision. Therefore, using the F1 score (0.95) provides a more balanced evaluation of model performance in cases with significant sample imbalance. In contrast, accuracy can be misleading in imbalanced datasets, as it may overestimate performance by favoring the majority class.

In this study, we used five different machine learning algorithms, achieving AUC values from 0.75 to 0.95. Initially, the inclusion of only statistically significant features (*P* < 0.05) yielded a lower AUC, as these variables alone did not capture the complexity of interactions affecting PND risk. Adding statistically insignificant variables helped the model account for subtle relationships, improving performance. Specifically, adding variables such as dexmedetomidine usage, anesthesia duration, and surgery duration (*P* > 0.05) improved the model's AUC.

To evaluate potential multicollinearity, we computed GVIFs for the retained non-significant variables. All GVIF^∧^(1/2Df) values were below 2, except for anesthesia duration (4.30), which was retained due to its clinical relevance. As AdaBoost is a tree-based method inherently robust to multicollinearity, model integrity was not compromised (Supplementary Table S2). Additionally, we conducted a likelihood ratio test comparing nested logistic regression models—one with only significant variables and another including all 22 features. While the full model showed reduced residual deviance, the difference was not statistically significant (*P* = 0.136). Nonetheless, these variables may enhance non-linear models like AdaBoost by capturing complex interactions and improving predictive performance.

Research shows dexmedetomidine reduces perioperative neurocognitive disorders by inhibiting inflammation and enhancing neuroprotection in older surgical patients and animal models (20). Dexmedetomidine, being negatively correlated with PND, helps to better differentiate between patient outcomes, thus improving the model's accuracy. Additionally, surgical trauma and prolonged anesthesia significantly increase PND and neuroinflammation risks (21, 22), suggesting that using a diverse set of variables could help uncover the intricate relationships between different factors (23).

On the other hand, the potential multicollinearity among feature variables could render the model parameter estimates unstable (24). Additionally, including more variables increases model complexity, which may improve data fitting but also heightens the risk of overfitting. To reduce overfitting and improve stability, we applied ten-fold cross-validation and adjusted parameters to ensure consistent model performance (25, 26).

Results showed the AdaBoost model achieved high AUC and accuracy in internal tests. This efficacy stems from AdaBoost's ability to handle small, imbalanced datasets effectively (27). However, concerns about the generalizability of models trained solely on internal data remain. Thus, collecting and evaluating external datasets is important for thoroughly assessing the model's predictive accuracy. Selecting features carefully is also vital for developing reliable predictive models.

In summary, despite challenges related to data imbalance and the diverse characteristics of older surgical patients, our findings demonstrate that machine learning can effectively build models to predict PND. By addressing class imbalances, incorporating sarcopenia-related indicators, and improving model accuracy and generalizability, we have developed a model with strong predictive power. This model provides a reliable risk assessment tool for older surgical patients, particularly those at risk due to sarcopenia.

Nevertheless, the present study has several limitations. Although the AdaBoost model achieved excellent performance (AUC = 0.95), potential overfitting cannot be ruled out due to the relatively small sample size and single-center data source. While 10-fold cross-validation and parameter tuning were employed to mitigate this risk, the model may still capture site-specific patterns, limiting generalizability. Additionally, sarcopenia-related features such as grip strength and muscle mass require standardized measurement protocols, which may not be uniformly available across institutions. Future multicenter and prospective validation is needed to confirm model robustness and clinical applicability.

Traditional machine learning algorithms are often critiqued for lacking transparency and interpretability (28). To address this issue, we applied SHAP values to explain our model's predictions, focusing on the AdaBoost model. SHAP values were used to determine how each feature influenced the model's output, highlighting important variables such as maximum grip strength, preoperative MMSE score, 6-meter walking speed, age, appendicular skeletal muscle mass, and sarcopenia assessment. These variables are crucial for tailoring perioperative strategies for older surgical patients.

Previous research indicates that grip strength correlates with cognitive declines in older adults (29). Our findings further suggest that stronger grip strength in elderly individuals is inversely associated with the risk of PND and cognitive impairment, whereas weaker grip strength is significantly linked to the onset of dementia and delirium. This association may stem from shared neural mechanisms between motor and cognitive functions (30). For instance, diminished grip strength may indicate systemic inflammation, a condition confirmed to correlate with cognitive decline, encompassing delirium and dementia. A decrease in preoperative grip strength not only reflects diminished muscle strength but may also signal dysfunction in related muscle groups, leading to impaired physical function and mobility, thereby increasing the risk of PND (10). Such patients are also found to have higher levels of postoperative inflammatory markers (S-100β, IL-6) (31).

Multiple studies have demonstrated that appendicular skeletal muscle mass is a key indicator of sarcopenia and muscle health, showing strong correlations with PND incidence: (1) Liu et al. reported that patients with reduced skeletal muscle mass exhibited significantly poorer performance on postoperative cognitive function tests, suggesting that low skeletal muscle mass is a potential risk factor for PND (1). (2) The reduction in skeletal muscle mass is associated with insulin resistance (32), energy metabolism disorders, increased inflammatory responses, oxidative stress, and cerebrovascular disease. These factors contribute to amyloid-beta deposition, tau protein hyperphosphorylation, and reduced neural plasticity (33). (3) Sarcopenic patients may experience changes in body composition, such as decreased lean body mass and increased fat, which can lead to dysfunction in organ systems, thereby altering the pharmacodynamics and pharmacokinetics of anesthetic drugs and resulting in lingering side effects that impair neural function.

The 6-meter walking speed is a reliable indicator of functional capacity and endurance in older individuals, correlating reductions with cognitive decline and increased depression, highlighting its role in diagnosing and managing sarcopenia (34). Studies have shown that a walking speed of < 0.8 meters per second in the 6-meter walking speed suggests the presence of sarcopenia (34). These measures serve not only as diagnostic criteria for sarcopenia but also reflect the overall health and functional status of patients. The connection between sarcopenia and PND indicates that patients with sarcopenia may be at greater risk for poor cognitive and physical recovery after surgery (35). Therefore, effective preoperative management of sarcopenia could improve outcomes and reduce the incidence of PND.

Age significantly influences the risk of PND, with a study of 1,064 patients showing higher prevalence in the elderly (36). Animal research also confirms that older mice are more prone to cognitive decline and Alzheimer's disease-related changes post-surgery, linked to β-amyloid accumulation (37). The risk of PND in older surgical patients is positively correlated with age, and this risk is further exacerbated by sarcopenia, which affects muscle mass and strength. By identifying these variables, we can improve the precision of PND risk prediction. To mitigate this risk, targeted preoperative interventions such as strength training and nutritional support could be implemented, potentially reducing the risk of PND and enhancing both cognitive and physical recovery after surgery.

## 5 Conclusion

In this study, we leveraged key variables, particularly those indicative of sarcopenia, to develop a suite of machine learning models that effectively predict the incidence of PND among the elderly. Specifically, the AdaBoost model demonstrated superior performance. Additionally, we applied the SHAP method to address the “black box” nature of machine learning, enabling us to determine the significance of each feature within our models. These models not only enable clinicians to identify older surgical patients more accurately at high risk for PND, particularly those with sarcopenia-related characteristics, but also aid in devising personalized perioperative management strategies that could significantly enhance patients' postoperative quality of life.

## Data Availability

The data analyzed in this study is subject to the following licenses/restrictions: The data that support the findings of this study are available from the first author upon reasonable request. Requests to access these datasets should be directed to Zhengyu Qian, 18384970837@163.com.
