# Correction: Application of machine learning in predicting perioperative neurocognitive disorders in elderly patients: the impact of sarcopenia-related features

**DOI:** 10.3389/fmed.2025.1690396

**Published:** 2025-09-03

**Authors:** Zhengyu Qian, Xiaochu Wu, Kunyang He, Kaijie Lin, Xiaobei Luo, Tianyao Zhang

**Affiliations:** ^1^School of Clinical Medicine, Chengdu Medical College, Chengdu, China; ^2^The First Affiliated Hospital of Chengdu Medical College, Chengdu, China; ^3^National Clinical Research Center for Geriatrics, West China Hospital, Sichuan University, Chengdu, Sichuan, China; ^4^Suzhou Medical College, Soochow University, Suzhou, Jiangsu, China

**Keywords:** machine learning, sarcopenia, postoperative cognitive dysfunction, SHapley Additive exPlanations, perioperative neurocognitive disorders

Affiliation “^2^The First Affiliated Hospital of Chengdu Medical College, Chengdu, China” was omitted for author Zhengyu Qian.

The conflict of interest statement is unchanged.

The original version of this article has been updated.

