# Supplementary figures and images for: Application of machine learning in predicting perioperative neurocognitive disorders in elderly patients: the impact of sarcopenia-related features
Source: Front Med (Lausanne). 2025 Aug 18;12:1604333. doi: 10.3389/fmed.2025.1604333 (PMC12399672; doi:10.3389/fmed.2025.1604333)

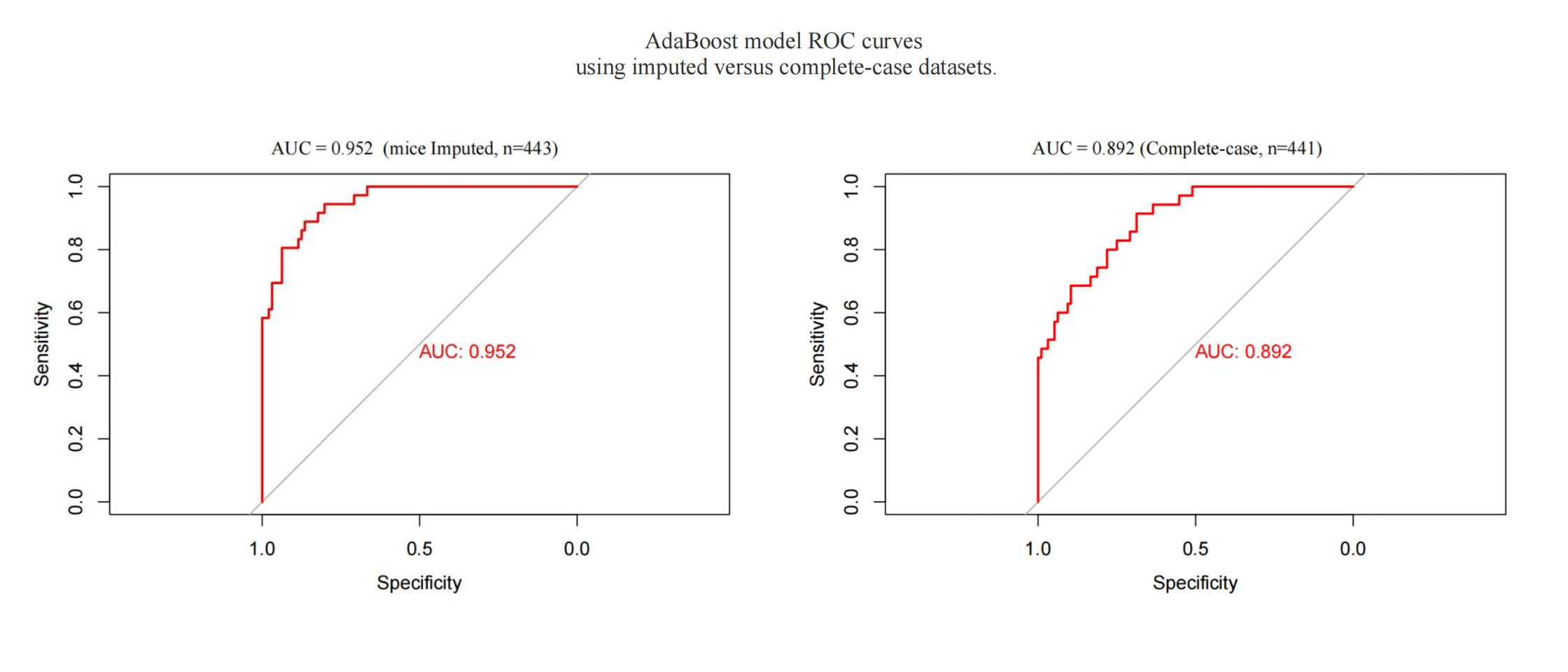

Supplement: Supplementary file 1 [file Image_1.jpeg]
